# Renal iron accumulation occurs in lupus nephritis and iron chelation delays the onset of albuminuria

**DOI:** 10.1038/s41598-017-13029-4

**Published:** 2017-10-09

**Authors:** Eileen S. Marks, Mathilde L. Bonnemaison, Susan K. Brusnahan, Wenting Zhang, Wei Fan, Jered C. Garrison, Erika I. Boesen

**Affiliations:** 1 Department of Cellular and Integrative Physiology, University of Nebraska Medical Center, Omaha, NE 68198 USA; 2 Department of Pharmaceutical Sciences, University of Nebraska Medical Center, Omaha, NE 68198 USA

## Abstract

Proteins involved in iron homeostasis have been identified as biomarkers for lupus nephritis, a serious complication of systemic lupus erythematosus (SLE). We tested the hypothesis that renal iron accumulation occurs and contributes to renal injury in SLE. Renal non-heme iron levels were increased in the (New Zealand Black x New Zealand White) F1 (NZB/W) mouse model of lupus nephritis compared with healthy New Zealand White (NZW) mice in an age- and strain-dependent manner. Biodistribution studies revealed increased transferrin-bound iron accumulation in the kidneys of albuminuric NZB/W mice, but no difference in the accumulation of non-transferrin bound iron or ferritin. Transferrin excretion was significantly increased in albuminuric NZB/W mice, indicating enhanced tubular exposure and potential for enhanced tubular uptake following filtration. Expression of transferrin receptor and 24p3R were reduced in tubules from NZB/W compared to NZW mice, while ferroportin expression was unchanged and ferritin expression increased, consistent with increased iron accumulation and compensatory downregulation of uptake pathways. Treatment of NZB/W mice with the iron chelator deferiprone significantly delayed the onset of albuminuria and reduced blood urea nitrogen concentrations. Together, these findings suggest that pathological changes in renal iron homeostasis occurs in lupus nephritis, contributing to the development of kidney injury.

## Introduction

Lupus nephritis is a serious complication of the autoimmune disease systemic lupus erythematosus (SLE), affecting up to 50% percent of patients^1^. Current treatments for lupus nephritis focus on immunosuppressive and anti-inflammatory approaches, which carry significant side effects and yet are sub-optimally effective, with remission slow or partial, and relapses common^2^. Accordingly, there is a clear need for novel therapies for lupus nephritis, and arguably, the search for new targets should extend beyond the immune system. To this end, studies describing urinary biomarkers of lupus nephritis may help identify suitable candidates.

Multiple proteins involved in iron homeostasis have been identified as urinary biomarkers of lupus nephritis or are increased in the urine of SLE patients with active disease. These include the iron-carrying proteins transferrin and neutrophil gelatinase associated lipocalin (NGAL or lipocalin-2)^3^, the iron storage protein ferritin^4^, ferroxidase enzyme ceruloplasmin^5,6^, and hepcidin^7^, which regulates gastrointestinal iron uptake and export of iron from cells. Increased circulating levels of ferritin have also been found in SLE patients with active disease^4,8^. These differences are noteworthy in that while iron is a key component of many proteins, disruption of iron homeostasis resulting in unbound redox active iron can promote cellular damage, apoptosis and ferroptosis via several mechanisms. These include production of the highly reactive hydroxyl radical by Fenton chemistry, lipid peroxidation, lysosomal injury, mitochondrial dysfunction and endoplasmic reticulum stress^9–12^. Recent studies have shown that dietary iron restriction or iron chelation reduce renal injury in mouse and rat models of diabetes, renal fibrosis and chronic kidney disease^13–15^, and increased dietary iron intake accelerates mortality and kidney damage in the MRL mouse model of SLE^16^. Despite these findings, whether iron accumulation occurs in the kidney in lupus nephritis, and the mechanisms by which this might occur have never been studied.

Multiple iron uptake pathways could contribute to renal iron accumulation. Most iron in the circulation is bound to transferrin, which in the kidney can be taken up via transferrin receptor 1 (TfR1), cubilin, and the NGAL receptor 24p3R^17–19^. Transferrin is a 78 kDa protein, (i.e., slightly larger than would be freely filtered at the glomerulus) and although some transferrin is normally present in the glomerular filtrate and is reabsorbed^19^, any degree of glomerular injury would enhance its filtration. “Free” iron is reabsorbed along the nephron^20^, with possible transporters including the divalent metal transporter 1 (DMT1)^21^, and divalent cationic metal transporters ZIP8 and ZIP14^22^. Reabsorption of other iron-carrying proteins such as ferritin, hemoglobin or NGAL by receptor-mediated endocytosis provide additional sources of iron^23^. Increased urinary excretions of transferrin, ferritin and NGAL in lupus nephritis patients^3–6^ provide evidence that the tubules of lupus nephritis patients have increased exposure to iron, which could facilitate renal iron accumulation and injury.

To test the hypothesis that iron accumulation occurs in and contributes to the development of renal injury in SLE, we studied renal tissue iron levels and uptake of iron prior to and around the onset of albuminuria in a well-established spontaneous model of lupus nephritis, the (New Zealand Black x New Zealand White) F1 mouse (hereafter NZB/W). New Zealand White (NZW) mice were included as age-matched healthy controls for comparison. We further tested whether treatment with a tissue-permeable iron chelator, deferiprone, blunts the development of albuminuria in the NZB/W mice. Our experiments focused on female mice, consistent with the marked female sex-bias observed in the prevalence of SLE in human populations (90% female)^24^.

## Results

### Renal iron accumulation occurs in the NZB/W mouse model of SLE

**Figure 1:**
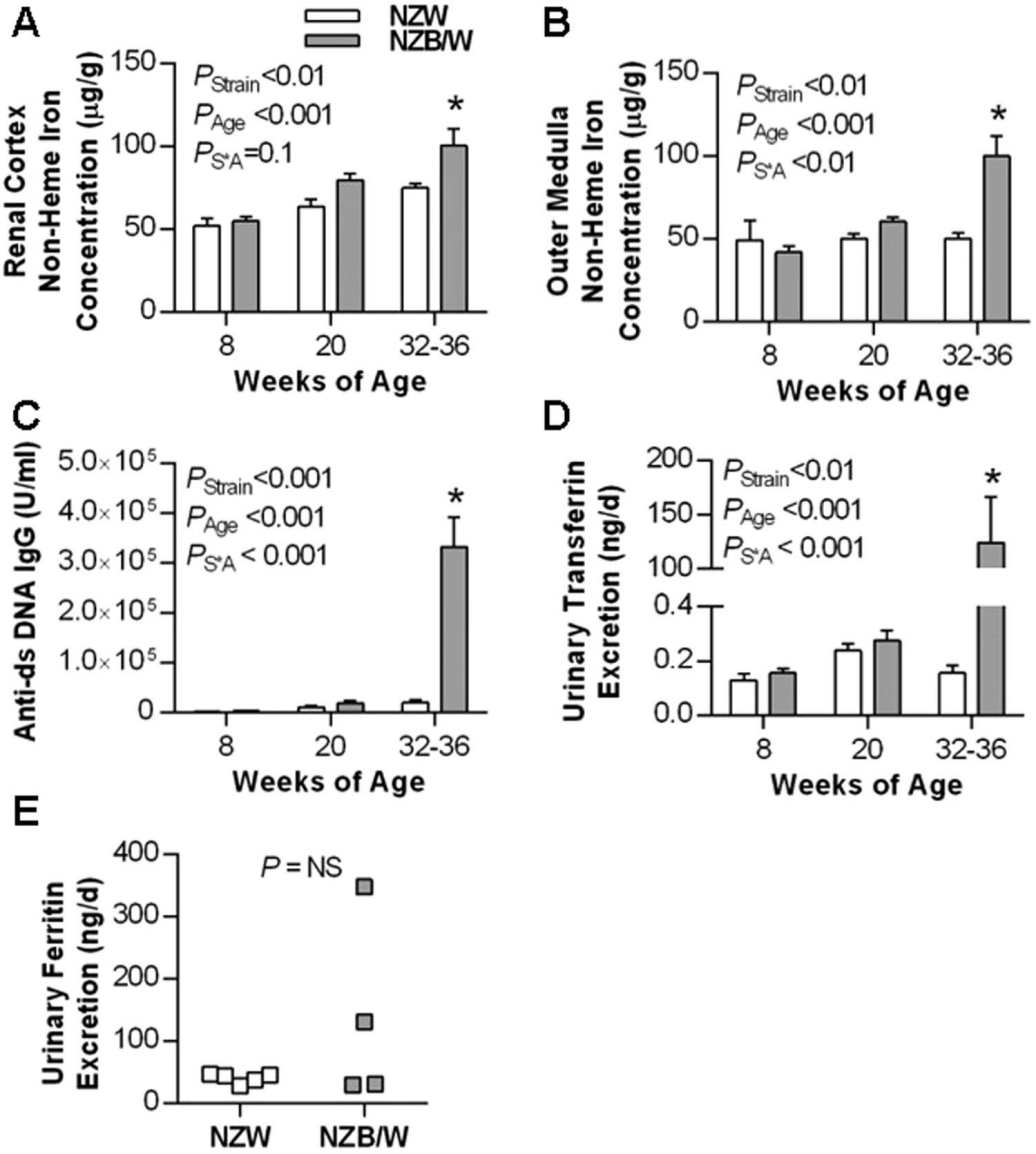
Renal non-heme iron levels are increased in the NZB/W model of SLE. NZB/W mice underwent weekly testing for albuminuria (defined as >2+ or >100 mg/dL in 24 h urine samples) and were sacrificed at the onset of albuminuria (32–36 weeks). Tissues and plasma from these mice were compared with age-matched NZW mice and 8 and 20 week old NZW and NZB/W mice. Non-heme iron concentration expressed per g wet tissue weight in (**A**) renal cortex and (**B**) outer medulla. (**C**) Anti-ds DNA IgG was measured in plasma. Mice were housed in metabolic cages for 24 h to allow sample collection for data depicted in (**D**) and (**E**). Urinary transferrin excretion for age-matched NZW and NZB/W mice (n = 4–6 per group) is presented in (**D**). Data other than in (**E**) are presented as mean ± SEM for n = 4–6 mice per group, with *P* values derived by two-way ANOVA, testing for main effects of mouse strain (*P*_Strain_), age (*P*_Age_) and whether concentrations changed with age differentially between strains (*P*_S*A_). *P < 0.05 compared to age-matched NZW mice. (**E**) Depicts urinary ferritin excretion in individual 32–36 week old NZW and NZB/W mice; data were compared by unpaired Student’s t-test.

Renal tissue non-heme iron concentrations, a measurement which excludes contributions from heme-containing proteins, were compared between NZB/W and NZW mice prior to and at the age of onset of albuminuria (32–36 weeks; determined by dipstick reading of >100 mg/dL). As shown in Fig. 1, non-heme iron concentrations in the renal cortex and outer medulla were similar between strains at 8 weeks of age, prior to the development of double-stranded (ds) DNA autoantibodies. By 20 weeks of age, renal iron levels were beginning to diverge and were significantly elevated in albuminuric NZB/W mice aged 32–36 weeks compared to age-matched NZW mice, at which time ds DNA autoantibodies were also strikingly elevated in the NZB/W mice. Plasma iron concentration was not significantly different between groups at 32–36 weeks of age, being 1.6 ± 0.3 µg/ml in NZB/W mice and 1.9 ± 0.1 µg/ml in NZW mice (*P* = 0.26). Hepatic mRNA expression of hepcidin, which inhibits egress of iron from duodenal enterocytes, was significantly reduced in 32–36-week-old NZB/W compared to NZW mice (2^−ΔΔCT^ of 0.33 ± 0.08 and 1.08 ± 0.19 respectively, *P* < 0.05).

### Urinary transferrin excretion and renal transferrin accumulation are increased in SLE

To investigate potential filtered sources of iron for re-uptake and accumulation by the kidneys, we measured the urinary excretion of two important protein carriers of iron: transferrin and ferritin. Urinary transferrin excretion was dramatically increased in albuminuric NZB/W mice (Fig. 1D). Urinary ferritin excretion was either below detection (<12.5 ng/ml) or approximately 20–40 ng/d for 8 and 20-week-old mice (data not shown) and variable in NZB/W mice at 32–36 weeks (Fig. 1E; *P* > 0.05 vs NZW). Attempts were made to measure total urinary iron excretion, however it appeared that these were confounded by the presence of small and variable amounts of food contamination in the metabolic cage urine collection tubes (data not presented).

**Figure 2:**
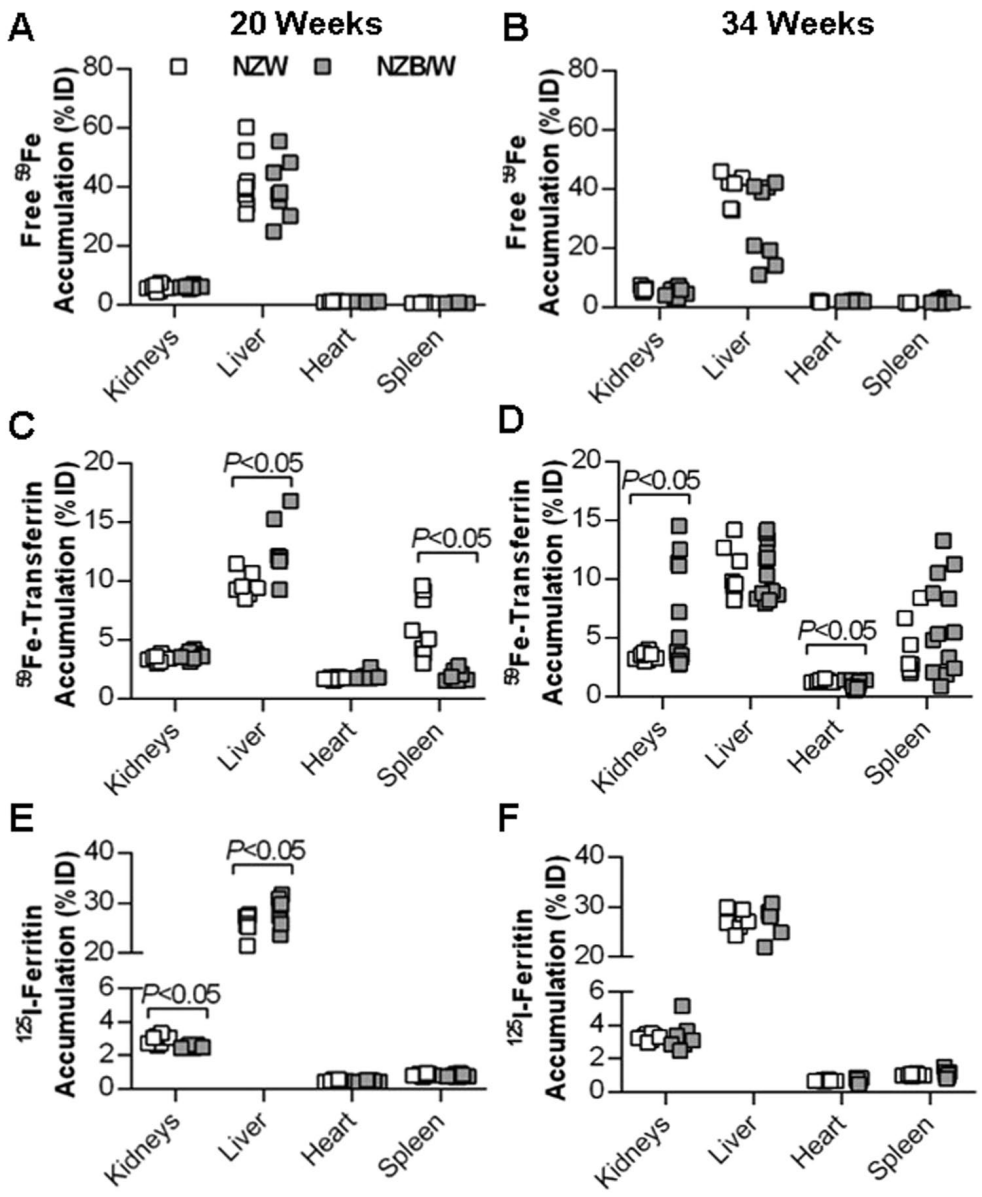
Biodistribution of iron and iron-binding proteins in select major organs following acute intravenous injection of radiotracers. Data from organs of individual mice, expressed as a percentage of total body radioactivity counts per minute (% of injected dose, %ID), are shown for 20 and 34 week old NZW and NZB/W mice, n = 7–13 per group. (**A** and **B**) Depict “free” (non-transferrin bound) iron distribution, with an injection of unlabeled iron citrate given prior to ^59^Fe to pre-saturate circulating transferrin. (**C** and **D**) Depict ^59^Fe-transferrin distribution, and (**E** and **F**) depict ^125^I-ferritin distribution. Groups were compared by Welch’s t-test (unequal variance t-test).

To investigate whether renal iron uptake in the form of “free” (non-transferrin bound iron), transferrin-bound iron or ferritin is increased in NZB/W mice, the biodistribution of acutely injected ^59^Fe(III) citrate, ^59^Fe-transferrin and ^125^I-ferritin was measured in NZW and NZB/W mice aged 20 weeks (healthy) or 34 weeks (median age of onset of albuminuria in NZB/W mice). The percentage of the total injected dose (%ID) that accumulated in the kidneys and several other organs and tissues was calculated. There were no significant differences between strains in free ^59^Fe accumulation by the kidneys or several other major organs regardless of age (Fig. 2A and B). The %ID of ^59^Fe-transferrin was significantly higher in the liver and lower in the spleen of 20-week-old NZB/W compared to NZW mice (*P* < 0.05), but %ID found in the kidneys was not significantly different (Fig. 2C). ^59^Fe-transferrin accumulation was, however, significantly increased in kidneys of NZB/W mice at 34 weeks (*P* < 0.05; Fig. 2D). ^59^Fe-Transferrin accumulation was slightly but significantly decreased in the hearts of NZB/W compared to NZW mice at 34 weeks (0.96 ± 0.09%ID versus 1.29 ± 0.04%ID respectively; *P* < 0.05; Fig. 2D). ^125^I-Ferritin accumulation was slightly but significantly lower in the kidneys and higher in the livers of NZB/W compared to NZW mice at 20 weeks (Fig. 2E), but no differences in %ID between groups were observed at 34 weeks (Fig. 2F).

**Figure 3:**
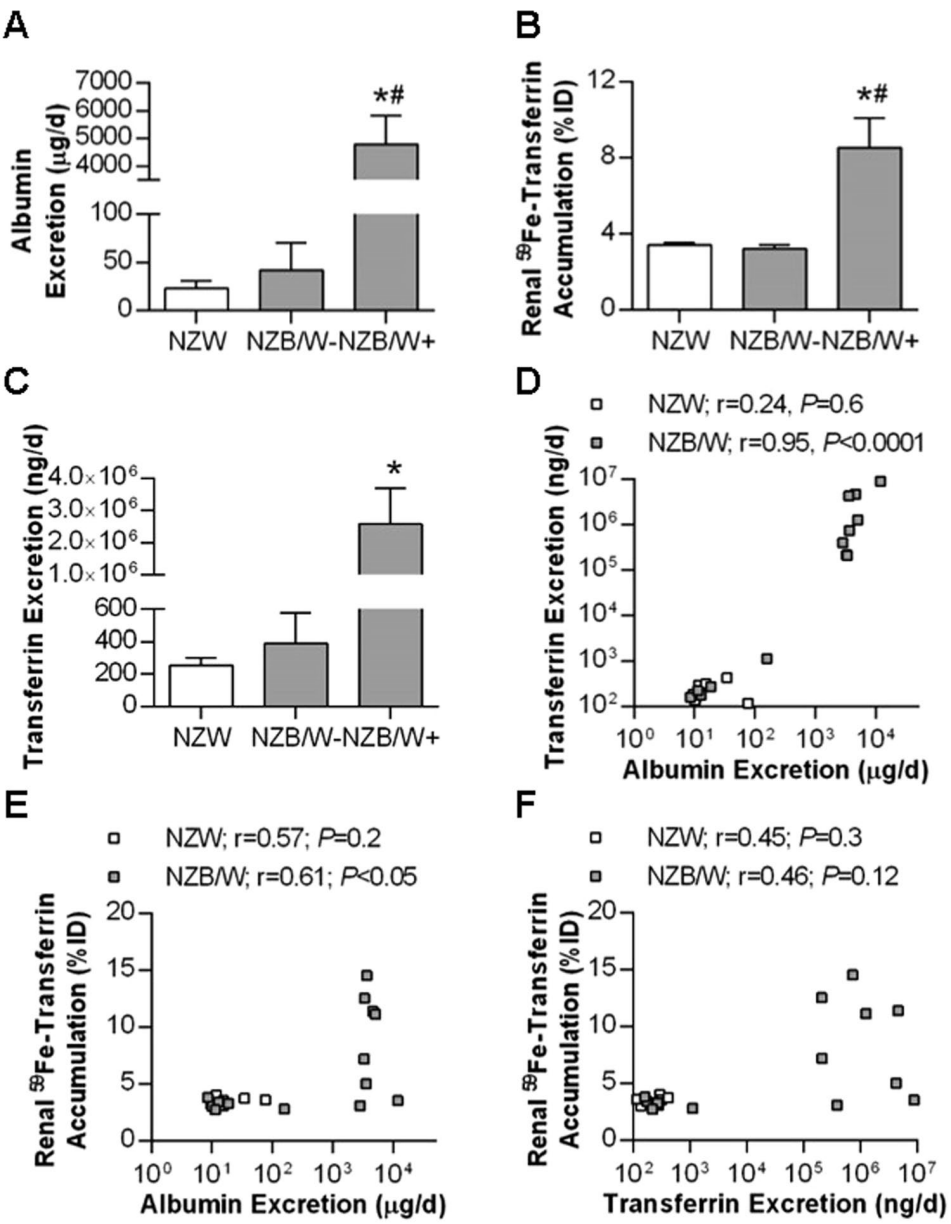
Comparison of renal transferrin handling in 34 week old albuminuric and non-albuminuric mice. NZB/W mice were subdivided into groups based on having urinary albumin concentrations of less than (NZB/W−; n = 5) or greater than 100 mg/dL (NZB/W+; n = 8), determined by ELISA. Comparisons were made between these two groups and age-matched NZW mice (n = 8). Group mean ± SEM are shown for (**A**) 24 h urinary albumin excretion, (**B**) renal ^59^Fe-transferrin accumulation (%ID) and (**C**) 24 h endogenous urinary transferrin excretion. Data points for individual NZW and NZB/W animals are plotted in (**D**–**F**), which depict the relationships between (**D**) rates of urinary albumin and transferrin excretion, (**E**) urinary albumin excretion rate and renal ^59^Fe-transferrin accumulation and (**F**) endogenous urinary transferrin excretion rate and renal ^59^Fe-transferrin accumulation. Data in (**A–C**) were compared by one-way ANOVA and Tukey’s post-hoc test where * indicates *P* < 0.05 vs NZW and ^#^ indicates *P* < 0.05 vs NZB/W−. Data in (**D–F**) were analyzed separately in NZW and NZB/W mice by Spearman’s rank-order correlation.

Increased renal ^59^Fe-transferrin accumulation by 34-week-old NZB/W mice could reflect enhanced filtration and reuptake of transferrin subsequent to changes in glomerular permeability. To test this further, the 34-week-old NZB/W mice were subdivided into albuminuric (>100 mg/dL; NZB/W+) and non-albuminuric (<100 mg/dL; NZB/W−) groups, and compared to the NZW mice (albumin excretions measured by ELISA are shown in Fig. 3A). ^59^Fe-transferrin accumulation by the kidney expressed as %ID was significantly increased in the NZB/W+ group compared to both the NZW and NZB/W− mice (*P* < 0.05; Fig. 3B). A similar result was seen for %ID per gram of tissue, being 16.5 ± 3.9%ID/g in NZB/W+, 8.2 ± 0.2%ID/g in NZW and 7.2 ± 0.3%ID/g in NZB/W− (*P* < 0.05). Endogenous transferrin excretion was significantly elevated in NZB/W+ compared to NZW mice (Fig. 3C). No significant differences in renal ^59^Fe-transferrin accumulation or urinary transferrin excretion were observed between NZW and NZB/W- mice (Fig. 3B and C). Correlation analyses were performed, showing a strong (r = 0.95) and significant (*P* < 0.0001) positive correlation between urinary excretion of albumin and transferrin in NZB/W mice but not in NZW mice (Fig. 3D). There was also a significant correlation between ^59^Fe-transferrin accumulation and albumin excretion in NZB/W but not NZW mice (Fig. 3E). The correlation between ^59^Fe-transferrin accumulation and endogenous transferrin excretion did not reach statistical significance in either group, however the NZB/W mice that displayed high %ID of ^59^Fe-transferrin in the kidneys uniformly also displayed high urinary excretion of endogenous transferrin (Fig. 3F).

In 34-week-old NZB/W and NZW mice that underwent free ^59^Fe biodistribution analysis, there was no significant correlation in either group between urinary excretion of albumin and %ID of ^59^Fe in the kidneys (Supplementary Figure 1). Although the numbers of albuminuric mice were more limited in the NZB/W cohort of 34-week-old mice used in ^125^I-ferritin distribution experiments (3 out of 8 were >100 mg/dL), significant positive correlations were observed between urinary ferritin and albumin excretion in both NZB/W and NZW mice (Supplementary Figure 2A), but not between albumin excretion and %ID of ^125^I-ferritin found in the kidneys (Supplementary Figure 2B), or between endogenous ferritin excretion and %ID of ^125^I-ferritin found in the kidneys (Supplementary Figure 2C).

### Tubular expression of iron handling proteins at 34 weeks

**Figure 4:**
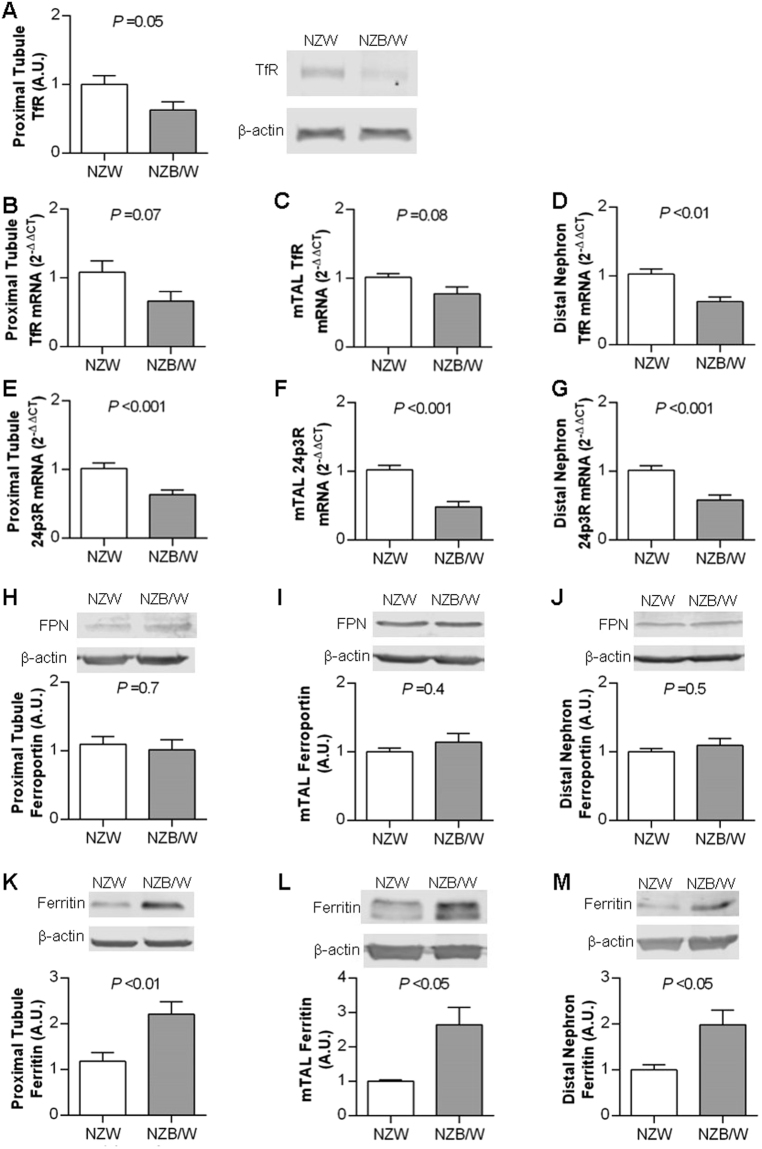
Expression of iron handling proteins in tubular preparations from 34 week old NZW and NZB/W mice. Proximal and distal nephron tubules were obtained following collagenase digestion and Percoll gradient fractionation of renal cortex. Medullary thick ascending limbs (mTAL) were obtained by collagenase digestion and sieving of outer medullary tissue. Tissue from 3–4 mice were pooled to generate each sample, with data shown as mean ± SEM for n = 8–13 samples per group. In all cases expression of the protein of interest was calculated relative to β-actin and the data normalized to the mean value of the NZW group. (**A**) Representative Western blot image and group analysis of proximal tubule transferrin receptor (TfR) expression. (**B**–**D**) Quantitative real-time PCR analysis of TfR mRNA expression in (**B**) proximal tubule, (**C**) mTAL and (**D**) cortical distal nephron preparations. (**E–G**) Quantitative real-time PCR analysis of24p3R mRNA expression in (**E**) proximal tubule, (**F**) mTAL and (**G**) cortical distal nephron preparations. (**H**–**J**) Representative Western blot images and group analysis of ferroportin (FPN) protein expression in (**H**) proximal tubule, (**I**) mTAL and (**J**) cortical distal nephron preparations. (**K–M**) Representative Western blot images and group analysis of ferritin protein expression in (**K**) proximal tubule, (**L**) mTAL and (**M**) cortical distal nephron preparations. Groups were compared by Student’s t-test.

The above data suggest that increased uptake of filtered ^59^Fe-transferrin may contribute to renal iron accumulation in SLE. Expression of two potential transferrin uptake pathways (TfR1 and 24p3R) were analyzed in kidney tissue preparations enriched for either proximal tubules, cortical distal tubules/collecting ducts or medullary thick ascending limbs (mTALs). TfR1 protein expression was significantly reduced in the proximal tubule of NZB/W compared to NZW mice (Fig. 4A); we were unable to accurately quantify TfR1 in mTALs and distal nephron-enriched preparations due to problems with background staining (data not shown). At the mRNA level, there was a non-significant trend for TfR1 expression to be lower in proximal tubules and mTALs of NZB/W mice compared to NZW mice (Fig. 4B and C). TfR1 mRNA levels in the distal nephron were significantly lower in NZB/W mice compared to NZW mice (Fig. 4D). Expression of 24p3R was significantly lower in NZB/W mice compared to NZW mice for all three preparations (Fig. 4E–G). Such findings would be consistent with compensatory downregulation at the mRNA stability level via iron-responsive proteins^25^ or potentially through increased ubiquitylation and lysosomal degradation^26^. Protein expression of the cellular iron efflux protein ferroportin in tubular preparations was not significantly different between strains (Fig. 4H–J), but levels of ferritin were approximately doubled in NZB/W compared to NZW mice in all three tubular preparations (Fig. 4K–M), consistent with increased intracellular iron storage in the NZB/W mice.

### Iron contributes to the development of albuminuria in SLE

**Figure 5:**
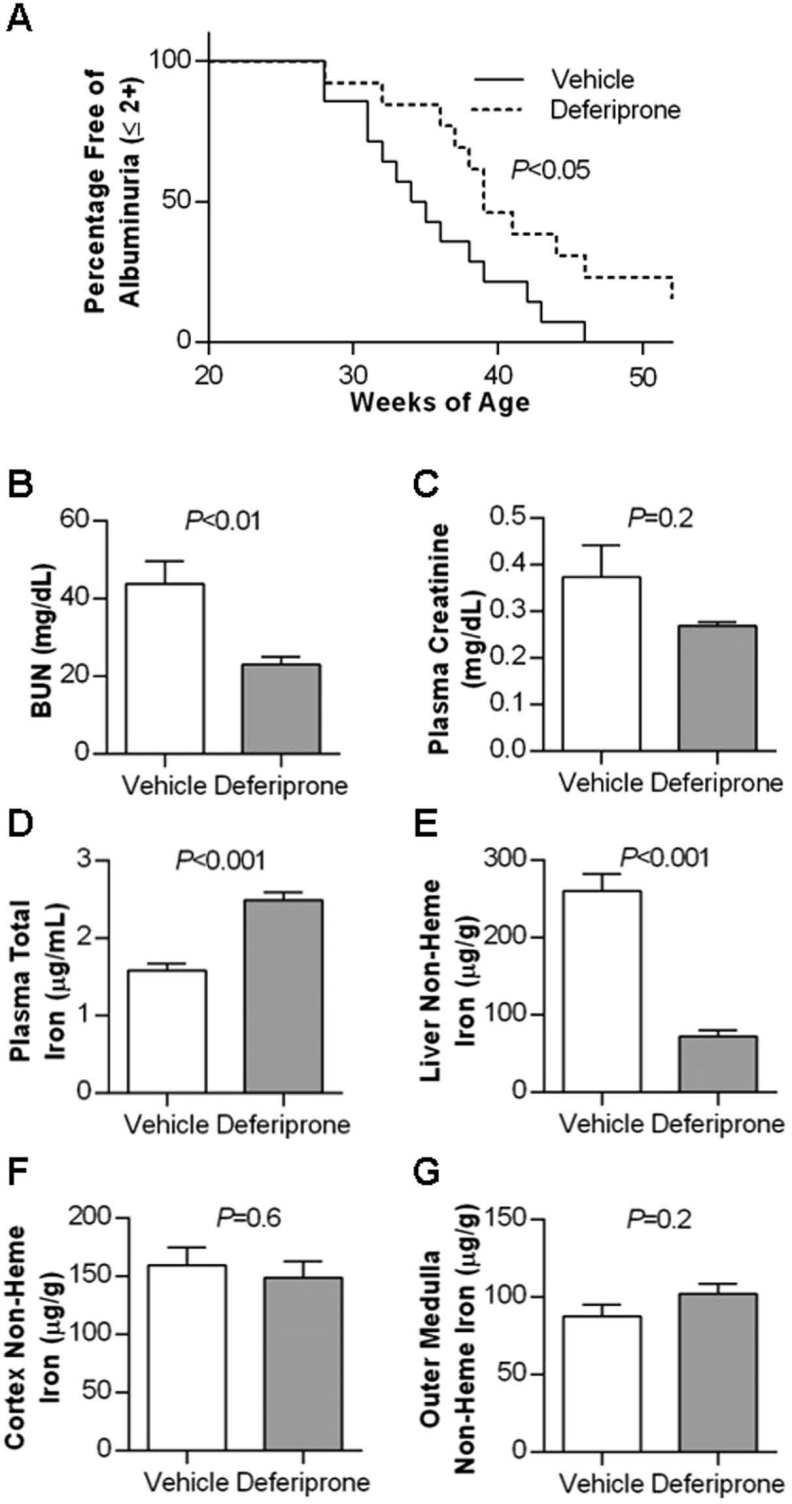
Effect of deferiprone treatment on the onset of albuminuria and body iron parameters in NZB/W mice. Treatment of mice with vehicle (n = 14) or deferiprone (n = 11) commenced at 20 weeks of age. The onset of albuminuria was monitored by dipstick with a reading of >2+ or >100 mg/dL considered positive for albuminuria, and the percentage of mice in each group remaining free of albuminuria up to 52 weeks is depicted in (**A**), with survival curves compared by log-rank (Mantel-Cox) test. Two deferiprone-treated mice remaining albuminuria-free to 63 weeks of age were excluded from tissue analysis. (**B**–**G**) Depict group mean ± SEM for samples collected from n = 14 vehicle and n = 9 deferiprone-treated mice at the onset of albuminuria, with groups compared by Student’s t-test. Data shown are blood urea nitrogen (BUN; B) and plasma creatinine concentration (**C**), and iron concentrations in (**D**) plasma, (**E**) liver, (**F**) cortex and (**G**) outer medulla.

To test the role of iron in renal injury in SLE, NZB/W mice were treated with either the orally-bioavailable and cell-permeable iron chelator deferiprone (1 g/L in drinking water^27^) or vehicle from 20 weeks-of-age onwards. Deferiprone treatment significantly delayed the onset of albuminuria (*P* < 0.05; Fig. 5A), with the median age of onset being 34.5 weeks in vehicle-treated mice and 39 weeks in deferiprone-treated mice. Two deferiprone-treated mice remained albuminuria-free through 63 weeks of age, at which time they were sacrificed but their plasma and tissues excluded from further analyses. All other mice were sacrificed at the onset of albuminuria; plasma concentrations of anti-ds DNA IgG were similar in the two groups (1.1 ± 0.3 × 10^6^ U/ml and 1.6 ± 0.2 × 10^6^ U/ml in vehicle and deferiprone groups respectively, *P* = 0.2). Blood urea nitrogen (BUN) was significantly lower in the deferiprone-treated mice (*P* < 0.01; Fig. 5B), with a similar trend observed for plasma creatinine that did not reach statistical significance (Fig. 5C). Hematocrit was significantly lower in the vehicle-treated mice (42.9 ± 2.0%) compared with the deferiprone-treated mice (48.4 ± 0.9%; *P* < 0.05), with several mice in the vehicle group having quite low hematocrit (25.9, 34.0 and 34.3%). Red blood cell hemoglobin concentration was not significantly different between groups (971 ± 16 µM in vehicle, 940 ± 27 µM in deferiprone; *P* = 0.3), indicating that at this dosage, deferiprone treatment did not produce anemia. Plasma iron was significantly increased (*P* < 0.05; Fig. 5D) and liver non-heme iron decreased (*P* < 0.05; Fig. 5E) in deferiprone-treated mice, consistent with tissue iron being mobilized by deferiprone for excretion. Plasma hemoglobin concentration was not significantly different between vehicle (47.8 ± 11.7 µM) and deferiprone-treated groups (28.9 ± 6.6 µM, *P* = 0.2), providing no overt evidence of hemolysis in the deferiprone group, and together with the hematocrit data, arguing against hemolysis providing an alternative explanation for the increased plasma iron concentrations observed. Plasma ferritin concentration was not significantly different between the two groups (697 ± 69 ng/ml in vehicle and 771 ± 65 ng/ml in deferiprone-treated mice; *P* = 0.5). Renal cortical and outer medullary non-heme iron concentrations were not significantly different between deferiprone and vehicle-treated mice (Fig. 5F and G).

## Discussion

The current study shows that iron accumulation is increased within the kidneys of the NZB/W mouse model of lupus nephritis, and that iron contributes to the development of albuminuria. Further, we also showed that not only is urinary excretion of transferrin increased following the onset of albuminuria in NZB/W mice, but that renal accumulation of transferrin following acute injection is also increased. Together with biomarker evidence in human studies^3–8^, our data are consistent with the hypothesis that dysregulation of iron metabolism and enhanced exposure of the kidney to iron contributes to the development of renal injury in lupus nephritis.

Although not extensively studied, iron contributes to several forms of acute and chronic kidney disease. Uptake of hemoglobin, myoglobin and heme has been implicated in renal and specifically tubular injury in rhabdomyolysis^28^ and following hemolysis^29^, as well as following cardiovascular surgery^30,31^ and in sickle cell disease^32,33^. Parenteral iron preparations have dose-dependent toxic effects on renal tubular epithelial cells^34,35^, and a recent study of hemodialysis patients reported an association between decline in residual kidney function and iron saturation at baseline^36^. Dietary iron restriction or iron chelation with deferiprone attenuates the development of diabetic renal injury in several different rodent models^13,37–39^, and dietary iron restriction prevents progression of renal injury in the 5/6 nephrectomy model of chronic kidney disease^15^. Together with the current study, these data indicate that iron-mediated renal injury also occurs in conditions that are not intimately associated with hemolysis, and may represent an under-investigated potential driver of chronic kidney disease. While the results of the current study are promising, the therapeutic potential of deferiprone or other iron chelators in preventing histological evidence of renal injury, including of the glomerulus, or delaying the further progression of renal injury in SLE awaits more in-depth investigation.

Identifying aberrant modes of iron uptake or handling could facilitate development of more targeted therapies. Our findings indicate that renal transferrin-mediated iron accumulation was significantly increased following the onset of albuminuria, and suggest that this may occur at least in part through tubular reuptake of filtered transferrin. No decrease in tubular ferroportin expression was observed, although the finding that hepatic hepcidin mRNA was significantly reduced in 32–36 week old NZB/W compared to NZW mice is consistent with the potential for improper regulation of ferroportin and iron handling in lupus; further examination of this issue is beyond the scope of the current study. Of clinical relevance, urinary transferrin excretion is increased in both adult and pediatric lupus nephritis patients with active disease^4,6^, and was recently included in a combinatorial biomarker panel found to predict decline of renal function in lupus nephritis patients^40^. Increased urinary transferrin excretion has also been proposed as a biomarker of diabetic nephropathy in multiple studies of type 1 and type 2 diabetic patients (reviewed in)^41^. Accordingly, enhanced tubular uptake of transferrin-bound iron may contribute to the progression of renal injury in SLE and other diseases.

Whether iron promotes glomerular injury in lupus nephritis is a relevant question. We did not attempt to isolate glomeruli to measure iron uptake pathways due to the variety of cell types present, potentially complicating data interpretation. Deferiprone delayed the onset of albuminuria, but the experimental design (i.e. sacrificing the mice at different ages but equivalent albuminuria status) confounds an assessment of the effect of deferiprone on histological markers of glomerular injury. The plasma creatinine and BUN concentrations observed in the current study further indicate that the level of renal injury and dysfunction present in these animals was still relatively mild. Based on the promising initial findings reported herein, future studies specifically designed to provide an in-depth assessment of whether iron chelation reduces renal injury at both early and more advanced stages of lupus nephritis are warranted.

Several different methods are available to measure iron in tissues, including the non-heme iron assay used in the current study^42^, magnetic resonance imaging^43^, atomic absorption spectroscopy^44^, histological stains such as Pearls’ Prussian Blue^45^ and fluorescent probes for use in cell systems^46,47^. To our knowledge, none of these have been used to determine whether or not iron accumulation occurs in the kidneys of lupus nephritis patients. A critical question in terms of tissue injury, however, is whether increased amounts of unbound, redox-active “labile” or “catalytic” iron is present, and this pool represents a tiny fraction of all iron present in tissues^48^. Unfortunately most of the methodologies used to assess iron in tissues, ours included, do not specifically measure the labile iron pool, representing a limitation to our approach. Accordingly, lack of an observable decrease in kidney non-heme iron levels with deferiprone treatment in our study doesn’t preclude a reduction of labile iron levels, and the renoprotective effect of deferiprone supports a role for iron in mediating renal injury in lupus nephritis.

Our data in a translationally-relevant mouse model of lupus nephritis suggest that not only do proteins related to iron metabolism serve as urinary biomarkers for renal injury, but that iron itself may contribute to the development of renal injury in lupus nephritis. While awaiting further confirmation by more in-depth studies, including testing of effects of deferiprone on glomerular injury and renal functional decline at more advanced stages of SLE, our data support the hypothesis that iron represents a potential new therapeutic target in lupus nephritis.

## Concise Methods

### Animal care

All animal experiments were approved in advance by the University of Nebraska Medical Center’s Institutional Animal Care and Use Committee and conformed to the NIH Guide for the Care and Use of Laboratory Animals. Female NZW and NZB/W mice were purchased from the Jackson Laboratory (Bar Harbor, ME) and housed in groups of four in our animal facility on a 12-hour light/dark cycle with *ad libitum* access to standard diet (Teklad LM-485 mouse/rat sterilizable diet, Envigo) and drinking water or water containing deferiprone (1 g/L; Sigma-Aldrich, St. Louis, MO; replaced twice per week).

### Sample collection

Mice were placed in metabolic cages for 24 h periods and body weight, water and food intake, and urine production were recorded. Onset of albuminuria was determined using Albustix^®^ colorimetric dipsticks (Siemens Healthcare Diagnostics, Tarrytown, NY), with albuminuria defined as a reading of >100 mg/dL (>2+ on a 0–4 scale). Urine was stored at −80 °C until further testing. An initial cohort of NZB/W mice underwent weekly metabolic cage collections from 20 weeks onwards to determine the age of onset of albuminuria. NZW mice were then age-matched to this group, and those and additional cohorts of 8 and 20-week-old NZB/W and NZW mice underwent one 24 h metabolic cage collection period prior to sacrifice. Mice utilized in biodistribution studies underwent a single 24 h metabolic cage collection one week prior to sacrifice (i.e. at either 19 or 33 weeks of age). For the deferiprone treatment study, mice began weekly 24 h metabolic collections at 28 weeks of age until detection of albuminuria using dipsticks as defined above.

Mice were euthanized via isoflurane overdose followed by thoracotomy. A terminal blood sample was collected by cardiac puncture in experiments other than biodistribution and tubule isolation studies. After collecting a small blood sample in heparinized mylar^®^ wrapped hematocrit tubes (Drummond Scientific Company, Broomall, PA) for determination of hematocrit by centrifugation, blood was centrifuged, with plasma and a sample of red blood cells lysed 1:1 with deionized water stored separately at −80 °C prior to analysis. Kidneys were rapidly removed, decapsulated, dissected and snap frozen in liquid nitrogen and stored at −80 °C until further analysis. Other organs of interest were also removed and weighed; small samples of liver tissue were collected from certain groups of mice and frozen at −80 °C until further analysis.

### Renal tubule isolations

Kidneys from three to four mice were pooled per sample to ensure usable final yield. Cortical proximal and distal nephron tubules and mTALs were collected for each pooled sample.

#### Cortical Proximal and Distal Nephron Tubule Isolation

Proximal and distal tubule enriched fractions were isolated from whole mouse cortex based on previously described protocols^49,50^ with minor modifications. Briefly, renal cortices were dissected and cubed at 100 µm thickness using a McIlwain Tissue Chopper (The Mickle Laboratory Engineering Co. Ltd., Gomshall, England) and digested in Krebs-Henseleit Saline (KHS) containing 1 mg/mL collagenase type I (Worthington Biochemical Corporation, Lakewood, NJ). The resulting suspension was filtered through a 200 μm sieve and separated by centrifugation using a 50% Percoll density gradient (Sigma-Aldrich, St. Louis, MO) in KHS. Tubule fractions were collected from the gradient, washed with KHS, pelleted and frozen at −80 °C until analysis.

#### Medullary Thick Ascending Limb Isolation

Medullary thick ascending limbs were isolated from mouse outer medulla using a previously described protocol with minor modifications^51^. Outer medullas were dissected and the outer stripe was discarded. The inner stripe was cubed at 100 µm thickness using a McIlwain Tissue Chopper and digested in a 2 mg/mL collagenase solution. The suspension was washed with HBSS and filtered through a 200 μm sieve. Thick limbs were pelleted and frozen at −80 °C until analysis.

### Iron biodistribution studies

Acute biodistribution of “free” (non-transferrin bound) iron, transferrin-bound iron and ferritin-bound iron were measured separately in 20 and 34-week-old NZB/W and NZW mice based on previously described methods^52–54^. Briefly, 70 µg of unlabeled ferric citrate was injected via the tail vein to transiently saturate plasma transferrin, then 5 µCi of ^59^Fe-labelled ferric chloride, dissolved in citrate buffer, was injected i.v. (Perkin Elmer, Waltham, MA), and the mouse sacrificed 5 min later. Following injection, over 80% of the non-transferrin bound (free) iron is cleared from the blood within 30 s^52^. To quantify transferrin-bound or ferritin-bound iron uptake by tissues, mice were sacrificed 1 hour after i.v. injection of either 150 µg (3 µCi) of ^59^Fe-transferrin or 150 µg (2 µCi) of ^125^I-apo-ferritin. ^59^Fe-transferrin was synthesized in a manner analogous to previous reports^55^. Attempts to label ferritin with ^59^Fe were unsuccessful; therefore, ^125^I-apo-ferritin was prepared through oxidative iodination using well-established procedures^56^ (^125^I was obtained from Perkin Elmer; equine apo-ferritin containing both heavy and light chains was purchased from Sigma-Aldrich). Radiolabeled proteins were peak-purified and labeling confirmed by radio-HPLC prior to injection to ensure preparations were not contaminated with un-bound ^59^Fe or ^125^I. This 1 h time-frame has been previously shown to allow for clearance of injected ferritin from the blood and distribution to tissues^53^. A NaI(Tl) well detector was used to measure total body counts of radioactivity and radioactivity of individual organs and tissues of interest, with uptake of radiotracers by individual organs calculated as a percentage of the injected dose (%ID).

### Assays of iron and iron-binding proteins

Tissue non-heme iron concentration in renal cortex, outer medulla and liver was measured by a microplate colorimetric assay following acid extraction and reaction with bathophenanthroline reagent as described by Grundy *et al*.^42^ Ferric citrate (Avantor^TM^ Performance Materials, Center Valley, PA) was used to make iron standards for the assay. Iron concentrations were normalized to wet tissue weight. Plasma total iron was measured using the Quantichrom™ Iron Assay Kit (BioAssay Systems, Hayward, CA). BUN and plasma creatinine were measured using Quantichrom^TM^ urea and creatinine assays, respectively (BioAssay Systems). Commercially available ELISAs were used to measure urine concentrations of transferrin (ADI, San Antonio, TX), ferritin (abcam, Cambridge, UK; also used for plasma) and albumin (Exocell, Philadelphia, PA). Plasma autoantibodies were measured using a mouse anti-dsDNA IgG-specific ELISA kit (ADI). Hemoglobin concentrations were measured in lysed red blood cells and in plasma using Cayman Chemical’s Hemoglobin Colorimetric Assay Kit (Ann Arbor, MI).

### Analysis of protein expression

For Western blot analysis, tubule pellets were homogenized in a pH 7.4 buffer containing 50 mM Tris, 0.1 mM EDTA, 0.1 mM EGTA, 0.1% β-mercaptoethanol, and 10% glycerol with protease and phosphatase inhibitors. Suspensions were centrifuged at 10,000 rpm for 5 min and total protein of supernatant quantified by Bradford assay. Isolated protein was loaded on 10% or 12% polyacrylamide gels and transferred to Immobilon-FL polyvinyl difluoride membrane (Millipore, Billerica, MA). Blots were blocked in 1:1 Li-Cor Odyssey Blocking Buffer (Li-Cor Biosciences, Lincoln, NE) and tris buffered saline (20 mM Tris, 137 mM NaCl, pH adjusted to 7.6). After blocking, blots were incubated with primary antibodies overnight at 4 °C. Fluorescently tagged secondary antibodies and a Li-Cor Odyssey infrared imaging system were used to visualize immunoreactivity. β-actin was used as a loading control to normalize protein of interest data. 20 and 34 week samples were blotted separately. Ratios were expressed relative to the average of NZW samples on each blot. Primary antibodies used include mouse transferrin receptor (catalog number 13–6800 from Invitrogen, Carlsbad, CA), rabbit ferroportin (MTP11-A from ADI, San Antonio, TX), and rabbit ferritin (ab75973 from abcam, Cambridge, UK). The ferritin antibody recognizes both light and heavy chain ferritin. Due to their similar size (19–21 kDa) they are indistinguishable under our running conditions; accordingly the single band at the appropriate molecular weight was quantified. Mouse (A1978) or rabbit (A2066) anti-β-actin antibodies were used for normalizing protein of interest data (Sigma-Aldrich, St. Louis, MO). Secondary antibodies used were AF-680 goat anti-mouse IgG (A21058) and AF-790 goat anti-rabbit IgG (A11369) from Invitrogen.

For quantitative real-time PCR analysis of tubular and liver gene expression, RNA was extracted from tubule pellets or liver tissue using RNeasy Plus Mini kits (Qiagen, Valencia, CA), and a QuantiTect RT kit (Qiagen) used to synthesize cDNA from 1 µg of RNA. Real-time PCR (RT-PCR) was performed using QuantiTect® SYBR® Green RT-PCR kits (Qiagen, Valencia, CA), QuantiTect® Primers Assays (Qiagen) and detected via a Rotor-Gene Q (Qiagen), with mRNA expression of genes of interest relative to β-actin calculated using the 2^−ΔΔCt^ method, normalized to the NZW group.

### Statistical analysis

For studies comparing NZB/W and NZW mice at different ages, data were analyzed by two-way analysis of variance (ANOVA), testing for main effects of mouse strain (*P*_Strain_), age (*P*_Age_) and whether the interaction between the two (*P*_S*A_), with Bonferroni post-hoc test to compare between strains at specific ages. Comparisons between three groups were performed by one-way ANOVA and Tukey’s post-hoc test. For comparisons between two groups, data were compared by unpaired Student’s t-test or Welch’s t-test (unequal variance t-test). Spearman’s rank-order correlation was used to test relationships between two variables in the biodistribution studies. Survival curves of the percentage of mice in vehicle- and deferiprone-treated groups remaining free of albuminuria were compared by log-rank (Mantel-Cox) test. Except were otherwise indicated, data are presented either as individual points or as group mean ± SEM, with *P* < 0.05 considered statistically-significant.

### Data availability

The datasets generated during and/or analysed during the current study are available from the corresponding author on reasonable request.

## Electronic supplementary material

Supplementary Figures

## References

[1] Houssiau FA (2010). The 10-year follow-up data of the Euro-Lupus Nephritis Trial comparing low-dose and high-dose intravenous cyclophosphamide. Ann Rheum Dis.

[2] Lightstone L (2010). Lupus nephritis: where are we now?. Curr Opin Rheumatol.

[3] Hinze CH (2009). Neutrophil gelatinase-associated lipocalin is a predictor of the course of global and renal childhood-onset systemic lupus erythematosus disease activity. Arthritis Rheum.

[4] Vanarsa K (2012). Inflammation associated anemia and ferritin as disease markers in SLE. Arthritis Res Ther.

[5] Suzuki M (2008). Neutrophil gelatinase-associated lipocalin as a biomarker of disease activity in pediatric lupus nephritis. Pediatr Nephrol.

[6] Suzuki M (2009). Initial validation of a novel protein biomarker panel for active pediatric lupus nephritis. Pediatr Res.

[7] Mohammed MF (2014). A Study of Hepcidin and Monocyte Chemoattractant Protein-1 in Egyptian Females With Systemic Lupus Erythematosus. J Clin Lab Anal.

[8] Pradhan V (2016). Association of Serum Ferritin Levels with Hematological Manifestations in Systemic Lupus Erythematosus Patients from Western India. J Assoc Physicians India.

[9] Huang ML, Lane DJ, Richardson DR (2011). Mitochondrial mayhem: the mitochondrion as a modulator of iron metabolism and its role in disease. Antioxid Redox Signal.

[10] Koskenkorva-Frank TS, Weiss G, Koppenol WH, Burckhardt S (2013). The complex interplay of iron metabolism, reactive oxygen species, and reactive nitrogen species: insights into the potential of various iron therapies to induce oxidative and nitrosative stress. Free Radic Biol Med.

[11] Lou LX (2009). Endoplasmic reticulum stress involved in heart and liver injury in iron-loaded rats. Clin Exp Pharmacol Physiol.

[12] Lu JJ, Chen SM, Zhang XW, Ding J, Meng LH (2011). The anti-cancer activity of dihydroartemisinin is associated with induction of iron-dependent endoplasmic reticulum stress in colorectal carcinoma HCT116 cells. Invest New Drugs.

[13] Ikeda Y (2013). Dietary iron restriction inhibits progression of diabetic nephropathy in db/db mice. Am J Physiol Renal Physiol.

[14] Ikeda Y (2014). Iron chelation by deferoxamine prevents renal interstitial fibrosis in mice with unilateral ureteral obstruction. PLoS One.

[15] Naito Y (2013). Dietary iron restriction prevents further deterioration of renal damage in a chronic kidney disease rat model. J Hypertens.

[16] Leiter LM, Reuhl KR, Racis SP, Sherman AR (1995). Iron status alters murine systemic lupus erythematosus. J Nutr.

[17] Kozyraki R (2001). Megalin-dependent cubilin-mediated endocytosis is a major pathway for the apical uptake of transferrin in polarized epithelia. Proc Natl Acad Sci USA.

[18] Langelueddecke C (2012). Lipocalin-2 (24p3/neutrophil gelatinase-associated lipocalin (NGAL)) receptor is expressed in distal nephron and mediates protein endocytosis. J Biol Chem.

[19] Zhang D, Meyron-Holtz E, Rouault TA (2007). Renal iron metabolism: transferrin iron delivery and the role of iron regulatory proteins. J Am Soc Nephrol.

[20] Wareing M, Ferguson CJ, Green R, Riccardi D, Smith CP (2000). *In vivo* characterization of renal iron transport in the anaesthetized rat. J Physiol.

[21] Canonne-Hergaux F, Gros P (2002). Expression of the iron transporter DMT1 in kidney from normal and anemic mk mice. Kidney Int.

[22] Galvez-Peralta M, Wang Z, Bao S, Knoell DL, Nebert DW (2014). Tissue-Specific Induction of Mouse ZIP8 and ZIP14 Divalent Cation/Bicarbonate Symporters by, and Cytokine Response to, Inflammatory Signals. Int J Toxicol.

[23] Martines AM (2013). Iron metabolism in the pathogenesis of iron-induced kidney injury. Nat Rev Nephrol.

[24] Beeson PB (1994). Age and sex associations of 40 autoimmune diseases. Am J Med.

[25] Wilkinson N, Pantopoulos K (2014). The IRP/IRE system *in vivo*: insights from mouse models. Front Pharmacol.

[26] Tachiyama R (2011). Proteome of ubiquitin/MVB pathway: possible involvement of iron-induced ubiquitylation of transferrin receptor in lysosomal degradation. Genes Cells.

[27] Song D, Song Y, Hadziahmetovic M, Zhong Y, Dunaief JL (2012). Systemic administration of the iron chelator deferiprone protects against light-induced photoreceptor degeneration in the mouse retina. Free Radic Biol Med.

[28] Panizo N, Rubio-Navarro A, Amaro-Villalobos JM, Egido J, Moreno JA (2015). Molecular Mechanisms and Novel Therapeutic Approaches to Rhabdomyolysis-Induced Acute Kidney Injury. Kidney Blood Press Res.

[29] Deuel JW (2016). Hemoglobinuria-related acute kidney injury is driven by intrarenal oxidative reactions triggering a heme toxicity response. Cell Death Dis.

[30] Billings FTt, Ball SK, Roberts LJ, Pretorius M (2011). Postoperative acute kidney injury is associated with hemoglobinemia and an enhanced oxidative stress response. Free Radic Biol Med.

[31] Qian Q, Nath KA, Wu Y, Daoud TM, Sethi S (2010). Hemolysis and acute kidney failure. Am J Kidney Dis.

[32] Vasavda N (2012). Renal iron load in sickle cell disease is influenced by severity of haemolysis. Br J Haematol.

[33] Wang Y, Doshi M, Khan S, Li W, Zhang PL (2015). Utility of Iron Staining in Identifying the Cause of Renal Allograft Dysfunction in Patients with Sickle Cell Disease. Case Rep Transplant.

[34] Zager RA, Johnson AC, Hanson SY (2004). Parenteral iron nephrotoxicity: potential mechanisms and consequences. Kidney Int.

[35] Zager RA, Johnson AC, Hanson SY, Wasse H (2002). Parenteral iron formulations: a comparative toxicologic analysis and mechanisms of cell injury. Am J Kidney Dis.

[36] Obi Y (2016). Residual Kidney Function Decline and Mortality in Incident Hemodialysis Patients. J Am Soc Nephrol.

[37] Matsumoto M (2013). Iron restriction prevents diabetic nephropathy in Otsuka Long-Evans Tokushima fatty rat. Ren Fail.

[38] Zou C (2017). Effect of the oral iron chelator deferiprone in diabetic nephropathy rats. J Diabetes.

[39] Zou C (2013). Iron chelator alleviates tubulointerstitial fibrosis in diabetic nephropathy rats by inhibiting the expression of tenascinC and other correlation factors. Endocrine.

[40] Abulaban KM (2016). Predicting decline of kidney function in lupus nephritis using urine biomarkers. Lupus.

[41] Matheson A, Willcox MD, Flanagan J, Walsh BJ (2010). Urinary biomarkers involved in type 2 diabetes: a review. Diabetes Metab Res Rev.

[42] Grundy MA, Gorman N, Sinclair PR, Chorney MJ, Gerhard GS (2004). High-throughput non-heme iron assay for animal tissues. J Biochem Biophys Methods.

[43] Wood JC (2015). Estimating tissue iron burden: current status and future prospects. Br J Haematol.

[44] Bush VJ, Moyer TP, Batts KP, Parisi JE (1995). Essential and toxic element concentrations in fresh and formalin-fixed human autopsy tissues. Clin Chem.

[45] Meguro R (2007). Nonheme-iron histochemistry for light and electron microscopy: a historical, theoretical and technical review. Arch Histol Cytol.

[46] Breuer W, Epsztejn S, Cabantchik ZI (1995). Iron acquired from transferrin by K562 cells is delivered into a cytoplasmic pool of chelatable iron(II). J Biol Chem.

[47] Hirayama T, Okuda K, Nagasawa H (2013). A highly selective turn-on fluorescent probe fro iron(II) to visualize labile iron in living cells. Chem Sci.

[48] Cabantchik ZI (2014). Labile iron in cells and body fluids: physiology, pathology, and pharmacology. Front Pharmacol.

[49] Vesey DA, Qi W, Chen X, Pollock CA, Johnson DW (2009). Isolation and primary culture of human proximal tubule cells. Methods Mol Biol.

[50] Vinay P, Gougoux A, Lemieux G (1981). Isolation of a pure suspension of rat proximal tubules. Am J Physiol.

[51] Yang J, Lane PH, Pollock JS, Carmines PK (2009). PKC-dependent superoxide production by the renal medullary thick ascending limb from diabetic rats. Am J Physiol Renal Physiol.

[52] Craven CM (1987). Tissue distribution and clearance kinetics of non-transferrin-bound iron in the hypotransferrinemic mouse: a rodent model for hemochromatosis. Proc Natl Acad Sci USA.

[53] Liu ZD, Lu SL, Hider RC (1999). *In vivo* iron mobilisation evaluation of hydroxypyridinones in 59Fe-ferritin-loaded rat model. Biochem Pharmacol.

[54] Wang CY, Knutson MD (2013). Hepatocyte divalent metal-ion transporter-1 is dispensable for hepatic iron accumulation and non-transferrin-bound iron uptake in mice. Hepatology.

[55] Hasan MR, Tosha T, Theil EC (2008). Ferritin contains less iron (59Fe) in cells when the protein pores are unfolded by mutation. J Biol Chem.

[56] Hermanson, G. T. in *Bioconjugate Techniques* Ch. 12, 515–517 (Academic Press, 1996).

