# Supplementary material for: Renal iron accumulation occurs in lupus nephritis and iron chelation delays the onset of albuminuria
Source: Sci Rep. 2017 Oct 9;7:12821. doi: 10.1038/s41598-017-13029-4 (PMC5634457; doi:10.1038/s41598-017-13029-4)

Supplementary Information for:

"Renal iron accumulation occurs in lupus nephritis and iron chelation delays the onset of albuminuria."

By

Eileen S. Marks[1], Mathilde L. Bonnemaison[1], Susan K. Brusnahan[2], Wenting Zhang[2], Wei Fan[2], Jered C. Garrison[2], Erika I. Boesen[1].

[1]Department of Cellular and Integrative Physiology, University of Nebraska Medical Center, Omaha NE 68198; [2]Department of Pharmaceutical Sciences, University of Nebraska Medical Center, Omaha NE 68198.

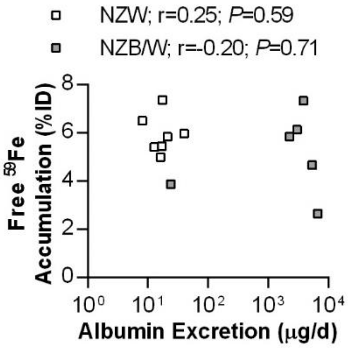

Supplementary Figure 1. Correlations between urinary excretion of albumin and renal free ^{59}Fe accumulation in 34 week old NZB/W and NZW mice. No significant correlation was observed in either group between urinary excretion of albumin and the %ID of ^{59}Fe found in the kidneys. Individual data points are shown for n = 7 NZW mice and n = 6 NZB/W mice.

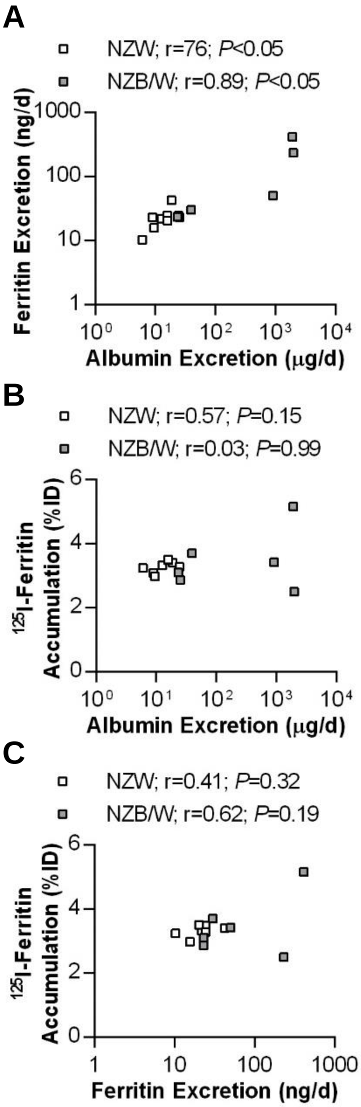

Supplementary Figure 2. Correlations between urinary excretion of ferritin or albumin, with renal ^{125}I-Ferritin accumulation in 34 week old NZB/W and NZW mice. (A) A significant correlation between urinary excretion of albumin and ferritin was observed in both groups (*P*<0.05 by Spearman correlation). No significant correlation was observed in either group between (B) urinary excretion of albumin and renal ^{125}I-Ferritin accumulation, or (C) urinary ferritin excretion and renal ^{125}I-Ferritin uptake. Individual data points are shown for n = 8 NZW mice and n = 7 NZB/W mice except for (C) where 1 out of 7 NZB/W mice from the ^{125}I-Ferritin bio-distribution study was not included due to insufficient urine for ferritin analysis.

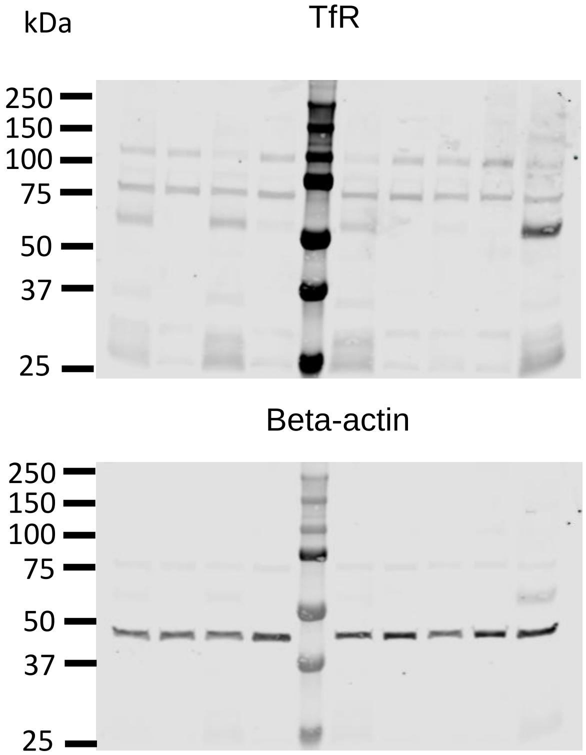

Supplementary Figure 3. Images of full length blot for TfR in enriched proximal tubule preparations. TfR (top) and beta-actin (bottom). The molecular weights of standards are indicated to the side of each image. The predicted molecular weight for TfR is 95kDa.

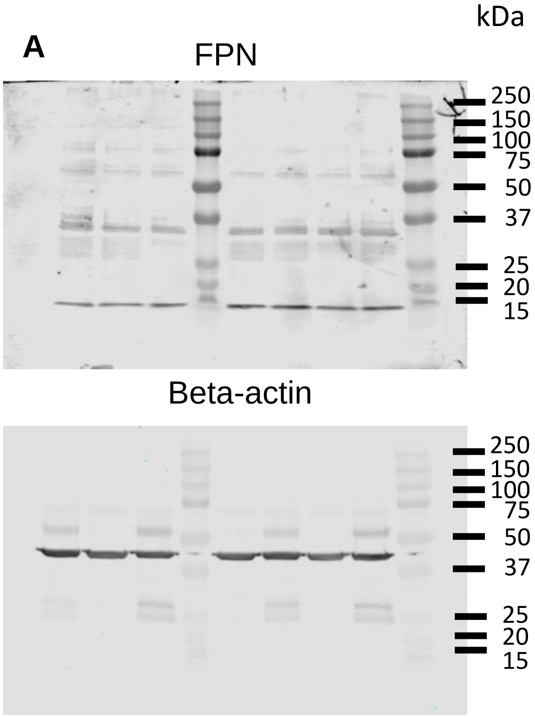

Supplementary Figure 4. Images of full length blots for FPN in enriched tubule preparations. The molecular weights of standards are indicated to the side of each image. The predicted molecular weight for FPN is 63 kDa. (A) FPN (top) and beta-actin (bottom) full length blots in proximal tubule enriched fractions. (B) FPN (top) and beta-actin (bottom) full length blots in thick limb enriched fractions. (C) FPN (top) and beta-actin (bottom) full length blots in distal nephron enriched fractions.

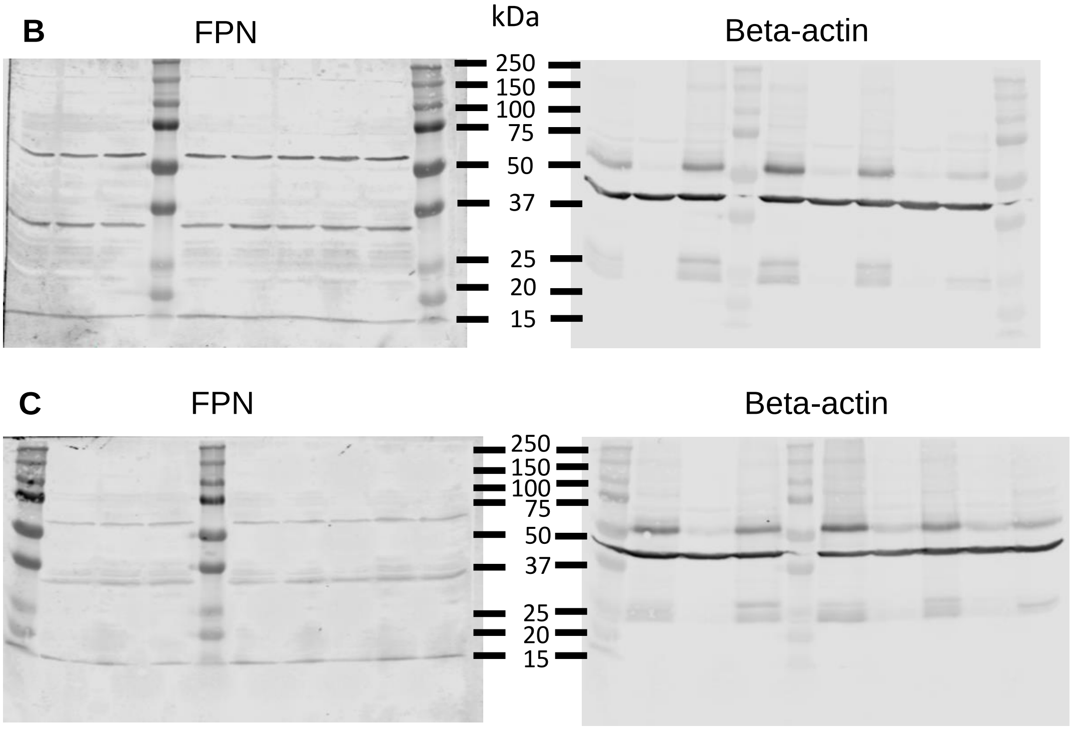

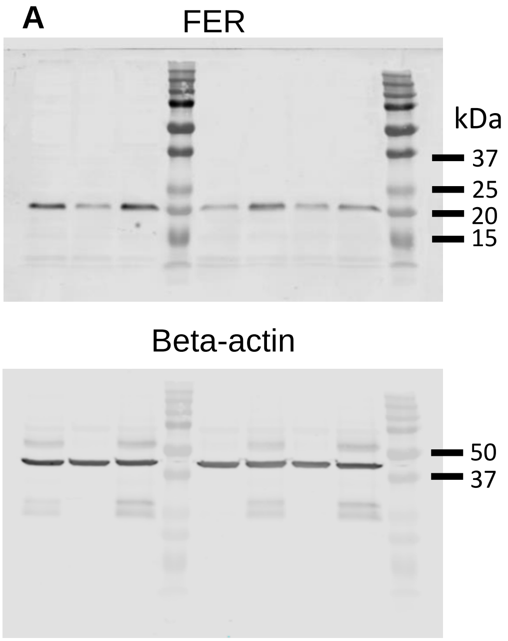

Supplementary Figure 5. Images of full length blots for FER in enriched tubule preparations. The relevant molecular weights of standards are indicated to the side of each image. Under our running conditions light and heavy chain FER are not distinguishable. The predicted molecular weight for FER is 19-21 kDa. (A) FER (top) and beta-actin (bottom) full length blots in proximal tubule enriched fractions. (B) FER (top) and beta-actin (bottom) full length blots in thick limb enriched fractions. (C) FER (top) and beta-actin (bottom) full length blots in distal nephron enriched fractions.

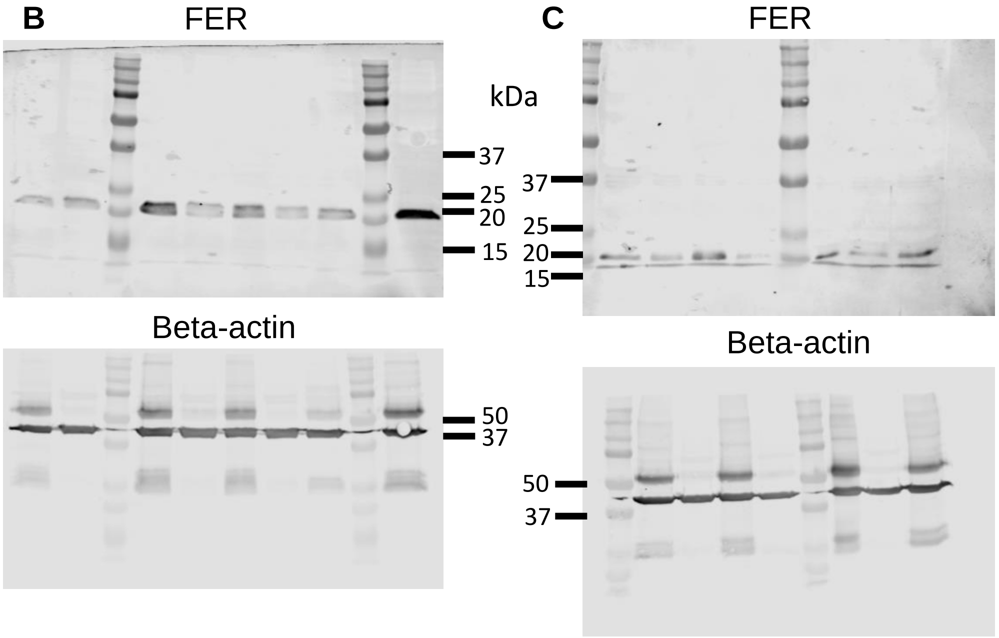

Supplement: Supplementary file 1 — Supplementary Figures [file 41598_2017_13029_MOESM1_ESM.pdf]
